# Dispensary personnel's views and experiences regarding oncologic cannabis and the counsel they offer adults with cancer

**DOI:** 10.1002/cam4.6634

**Published:** 2023-10-26

**Authors:** Manan M. Nayak, Peter R. Chai, Stephanie Tung, James A. Tulsky, Marilyn Hammer, Nicole Andrade, Ilana M. Braun

**Affiliations:** ^1^ Department of Psychosocial Oncology and Palliative Care Dana‐Farber Cancer Institute Boston Massachusetts USA; ^2^ Phyllis F. Cantor Center for Research in Nursing Dana‐Farber Cancer Institute Boston Massachusetts USA; ^3^ Harvard Medical School Boston Massachusetts USA; ^4^ Department of Emergency Medicine Brigham and Women's Hospital Boston Massachusetts USA; ^5^ The Fenway Institute Boston Massachusetts USA; ^6^ Department of Psychiatry Brigham and Women's Hospital Boston Massachusetts USA; ^7^ Department of Medicine Brigham and Women's Hospital Boston Massachusetts USA

**Keywords:** cannabis, complimentary therapies, dispensary personnel, medical oncology

## Abstract

**Background:**

A minority of oncologists feel qualified to advise adults with cancer on issues pertaining to medicinal cannabis. Adults with cancer frequently access medicinal cannabis information from non‐medical sources such as cannabis dispensaries. We explored dispensary personnel's views and experiences regarding oncologic cannabis and the counsel they extend individuals with cancer.

**Methods:**

Snowball sampling in this qualitative study facilitated recruitment across 13 states (*N* = 26). Semi‐structured phone interviews ceased with thematic saturation. A multi‐stage thematic analysis combined inductive and deductive codes.

**Results:**

Of the 26 dispensary personnel interviewed, 54% identified as female and 19% as non‐white. Median age was 40 years. A consensus emerged among participants concerning the botanical's efficacy for cancer‐related symptoms; less so regarding its antineoplastic potential. Principles for serving those with cancer included provision of client‐centered, symptom‐based, and trial‐and‐error approaches. Non‐inhalation modes of administration were generally recommended. No consensus was reached as to whether delta‐9‐tetrahydrocannabinal (THC)‐ or cannabidiol‐predominant products were preferable in this population. Challenges in oncologic advising included successfully identifying individuals with cancer at the dispensary counter, financial toxicity, the special treatment required for the THC‐naïve, and operating in the absence of standardized guidelines.

**Conclusions:**

These informed assertions suggest that members of the oncologic community should grapple with the extent to which they feel comfortable with both the nature and degree of counsel adults with cancer receive through dispensaries.

## Abstract 2

We interviewed dispensary employees (*N* = 26) to understand their cannabis therapeutics views and practices with oncologic populations. Participants agreed that cannabis is efficacious for cancer‐related symptoms; however, consensus was not reached regarding the botanical's antineoplastic potential. Care for adults with caner consisted of client‐centered and symptom‐based. Challenges in oncologic advising included successfully identifying individuals with cancer at the dispensary counter, financial toxicity, the special treatment required for the THC‐naïve, and operating in the absence of standardized guidelines.

## 1 BACKGROUND

Medicinal cannabis is legal in 38 states and the District of Columbia.^1^ A cancer diagnosis qualifies for the botanical in almost every such state law.^1^ Twenty‐three of these states (and the District of Columbia) have in place parallel non‐medical “adult use” cannabis laws which provide an additional avenue by which individuals with cancer may access cannabis.^1^ Although the true prevalence of medicating with cannabis in oncology is unknown, large convenience samples suggest that one out of every four or five individuals with cancer turn to cannabis during cancer treatment, mainly with medicinal intent.^2^, ^3^ Their rationales for medicating with cannabis include management of a variety of symptoms and cancer treatment side effects, and in pursuit of cancer‐directed therapy.^2^, ^3^, ^4^ Research indicates that a minority of oncologists feel qualified to clinically counsel adults with cancer regarding cannabis and that people with cancer access medicinal cannabis information from non‐medical sources, chiefly cannabis dispensaries.^4^, ^5^ Both quantitative and qualitative social–behavioral studies suggest that cannabis dispensary personnel tend to receive little or unstandardized on‐the‐job training in cannabis therapeutics, and may not counsel clients on some important risks including use disorders, psychosis and driving liabilities.^6^, ^7^, ^8^, ^9^ Collectively, these findings lead to a question about the nature of the advice offered to individuals with cancer at the dispensary counter. The following report examined semi‐structured interview data to explore dispensary personnel's views on adults with cancer medicating with cannabis, their experiences in offering advice to this population, the practical counsel they offer, and some of the challenges encountered.

## 2 METHODS

The researchers contacted state‐sanctioned cannabis dispensaries across 13 states (CA, CO, FL, IL, MA, NH, NV, NY, OH, PA, RI, VT, and WA) and cannabis dispensary personnel with whom they were familiar. The recruitment materials explained that the study sought to “understand the role marijuana plays in cancer care” and asked potential participants to share “thoughts and experiences with use of marijuana in cancer.” Between February 2020 and January 2021, snowball sampling identified 38 potential interviewees, of whom 11 were lost to follow‐up, one was ineligible, and 26 were audio‐recorded and thanked with $75 (68% response rate). Written consent was waived; however, elements of consent were reviewed. Eligibility criteria included: ≥18‐years of age, a US resident, English‐speaking, and in a client‐facing (e.g., sales, client education) and/or managerial (e.g., overseeing staff, staff training) role. Purposeful sampling procedures captured geographic and demographic diversity for both medical and non‐medical cannabis dispensary personnel.

Employing aspects of grounded theory, the principal investigator (IMB) and qualitative methodologists (IMB, MMN, and PRC) designed the semi‐structured interview guide which the multidisciplinary research team reviewed for clarity and thematic comprehensiveness. Investigators (IMB, MMN, and PRC) debriefed after the first five interviews and further refined the guide, namely including probes about varying state laws and their impacts on product availability. The interview guide was wide‐ranging in its exploration of indications for cannabis medicating. Cancer‐focused queries included why adults with cancer medicate with cannabis, whether the botanical works for those indications, and the types of advice participants deliver to clients with cancer at the dispensary counter.

Three trained qualitative researchers with cannabis content expertise (MMN, IMB, and PRC) conducted interviews. Based on debriefs with the research team after every few interviews, interviews ceased after no new emergent themes surfaced (i.e., thematic saturation was achieved).^10^ Two researchers (IMB and MMN) coded and analyzed transcripts using a multi‐stage thematic analysis, combining inductive and deductive codes, and incorporating aspects of grounded theory and applied framework analysis. A coding tree using interview guide domains provided an initial baseline framework. An inductive open coding approach was applied and emergent concepts added to the codebook. NVivo 12 (QSR International) facilitated coding. At the conclusion of coding, the study team discussed all themes to ensure consistency between data and findings. For these analyses, search terms from the transcribed interview data included: cancer, tumor, oncologist, oncology, chemotherapy, chemo, and Rick Simpson. The Dana‐Farber/Harvard Cancer Center Institutional Review Board approved study procedures. Methodology is additionally explained elsewhere.^6^

## 3 RESULTS

Of the 26 participants interviewed, over half (54%) identified as female and 19% as non‐White. Median age was 40 years. Forty‐two percent worked in a dispensary that exclusively sold medicinal cannabis, 38% in a dispensary that also offered “adult use” products. Half worked in a client‐facing role and half in a managerial one, sometimes with client‐facing responsibilities as well. Mean number of years in the cannabis industry was 5.27. Table 1 reports participant demographics and Table 2 exemplar quotations.

**TABLE 1:** Participant demographics.

	*n*	%
Mean age (Range)	26	40 (22–64)
Mean years in cannabis industry (Range)	5.27	(0.6 months–32 years)
Gender		
Female	14	54%
Male	11	42%
Other	1	4%
Ethnicity		
Non‐Hispanic/non‐Latino	22	85%
Hispanic/Latino	3	12%
Did not wish to report	1	4%
Race		
White	21	81%
Asian	2	8%
African American	1	4%
Native American	1	4%
More than one race	1	4%
Education		
Post‐graduate degree	12	46%
College graduate	11	42%
Some college	3	12%
Party affiliation		
Democrat	10	38%
Republican	5	19%
Independent	6	23%
Other/prefer not to answer	5	19%
Dispensary type		
Medicinal cannabis	11	42%
Medicinal and ‘adult use’	10	38%
‘Adult use’ cannabis	5	19%
Role at dispensary		
Client Facing	13	50%
Managerial	13	50%

**TABLE 2:** Exemplar Quotations.

Theme	Subthemes	Quotes
Common reasons adults with cancer medicate with cannabis		“For sure, we see over and over and over again how cannabis can help with the nausea, the vomiting, the appetite stimulation when folks are undergoing chemo – you know, pain and mood elevation. Let alone like neuroprotectant potential and things like that”
		“I think mostly they're not seeking it for the idea they'll shrink tumors. They're seeking it for the side‐effects of the chemotherapy and radiation, and also the depression and fear and anxiety that comes with a cancer diagnosis”
Cannabis's efficacy	For cancer‐related symptoms and side‐effects	“Complete 180. It was like night and day. So, then, these are people that were struggling, weren't able to get back out into society. Um, and then just by having this [cannabis] treating their symptoms, they were able to get things under control. Have their pain scores or nausea or the inability to‐to keep weight on. You know, the example of like, cancer patients?”
		“Two weeks later [my patient with endometrial cancer] came to see me and she was walking much straighter and she said that she was getting a great deal of relief… And her doctor said to her a few months later, I don't know what's happening – what you're doing, but for the first time in two years I'm seeing a change, and whatever you're doing, just keep doing it”
		“I do believe that cannabis is very necessary in cancer research and care just because I've seen the effects of chemotherapy on individuals and have seen that cannabis is one of the few if not the only substance that really counteracts a lot of those negative effects of chemotherapy”
		“How it helps certain ailments and things like cancer and just all sorts of ailments, how it can really help”
		“He's actually is in hospice, brain cancer and ‐ and liver… you know how morphine can be such a problem? It's a great pain medication, but, you know, the last days of someone's life, you don't want to not be able to communicate with'em”
		“She developed breast cancer and she did die from breast cancer eventually and was just on such heavy doses of morphine for the pain of metastasized bone cancer unfortunately that she was using medical marijuana. And what she told me was when she consumed medical marijuana, she could feel her soul again”
	For antineoplastic effects	“So I actually have had a couple of patients that have disclosed to me that they had or have cancer, and so one of them that always comes to mind is a patient of mine, she told me that she was on an [Rick Simpson Oil] protocol… She told me that she had 200 masses or lumps in her body of cancer and over a year or two's time after doing the [Rick Simpson Oil] protocol, all of her cancer was gone, and she told me she didn't do any chemotherapy or anything like that. Yeah. So that's pretty amazing. And she attributes it all to the [Rick Simpson Oil]”
		“I am a huge fan of [Rick Simpson Oil] for people that are going through any kind of cancer treatment, or they just got diagnosed, or for – even – I take [Rick Simpson Oil] every evening for preventative measures”
		“[Cannabis] helps with tumor shrinkage also. So there's a study in Spain that showed the tumor shrinkage info, and I think it's been replicated, but none of us are out there saying cannabis is shrinking tumors. But I certainly recommend that people look into that”
		“There's still a lot of folktale‐y things that go on. Or even, for example, when you're looking at using cannabis oil for a cancer therapy protocol – which is something we do not talk about at the shop. When people ask me about that, I refer them to people who are doing the research and the protocols because it's irresponsible for us to even have that conversation… When you're going into Rick Simpson and he's saying start with the size of a grain of rice – half a grain of rice and get up to a gram a day, well, you could possibly be overflooding your endocannabinoid system.
		“We took inbound calls. If they were about product [consumption], how to [consume] it, anything that didn't cross the line of should I [consume] this because I have cancer, or will this help my cancer?”
General principals in counseling adults with cancer	Client‐centered, symptom‐based approach	“The cancer patients tend to be very unique… it's challenging at times because you could have three or four different patients that have cancer have the same kind of cancer but when it comes down to treatment options, they each have different priorities”
		“Because cancer and cancer treatments can have a wide range of symptoms, so a lot of what we're dealing with is, all right, is it your nausea that's bad today, are you eating, is it your appetite that needs help, is it pain? So, again, a lot of what we target is the symptoms specifically. Not necessarily, oh, you're a cancer patient, this is specifically what we have for you”
	Trial‐and‐error	“I'm not going to say it's trial through error, but it's trial through experimentation. And oftentimes what we find out is – and this is particularly true with like some cancer patients – is that it's going to take several different iterations for you to get the desired effect”
Recommended modes of administration	Oral or sublingual typical	“Most cancer patients do not want any [inhaled] flower at all. They want tinctures, or RSO [Rick Simpson Oil], or edibles. And most edibles do not really have a terpene profile because once they get to a certain heating point, they burn off. So, for cancer patients, I always, always, always the first thing I suggest is [Rick Simpson Oil]. That's the first thing I suggest… Most of the time, they don't want to smoke”
		“[A friend with cancer] did not want to do any smoking, any inhaled, any type of that. So everything was either – it was more along the line of edibles or tinctures”
		“Because I always let them know, if they do it sublingually, it's going to hit their system a lot faster and the effects will – they'll feel it a little bit longer than if they were just doing an edible, I feel like. And I'm not 100 percent sure between the quickness. I feel like sublingual, it'll – it goes straight into your bloodstream so much faster, I feel like, than an edible would. So, those are the two routes that I go for cancer patients”
	Inhaled when rapid onset needed	“[Pertaining to cancer patients]: So I'm in really bad pain and I need that pain to go away quickly. Okay. Well, let's – you know, the quickest way of pain relief, the quickest onset of effect is going to be through inhalation”
Favored ingredient(s) for adults with cancer	Delta‐9‐tetrahydrocannabinol (THC)	“The reason we usually start at a one‐to‐one with a naive [cancer] patient is, you know, it ‐ it just gives you a nice starting point… If it's an all‐THC product, it may have some negative effects in terms of, you know, paranoia or intoxication… If a patient has neuropathy pain, we tend to [recommend] more THC… And if they call back and say, “Yeah, it's just not touching my neuropathy,” we'll then go up to a higher THC product”
		“[In reference to cancer patients]: THC, a lot of people are afraid of it. But it's actually really good for helping with pain, getting you eating. And one of the things that I see a lot when I work with physicians and nurses or when they recommend people come see me is that they're recommending CBD. And CBD is great, but I really feel like in many ways people recommend CBD because they know it's not going to get the patient high. And you know, people are afraid of euphoria”
	Cannabidiol (CBD)	“I had a patient that came to see me several years ago with endometrial cancer and she'd never [consumed] cannabis before, she was in a great deal of pain… I gave her an 18‐to‐1 CBD‐to‐THC concentrate and taught her how to parse it out so that she could [consume] it daily to help with pain management. We wanted to see how cannabis would work with her body since she was cannabis naïve”
		“I would say sometimes – the ones that – the people that were already using cannabis or accepting of cannabis, then they want to have more intoxicated feeling. But for the ones that are going through treatment, don't know what else to do, they don't have any other options, their doctor said that they should try cannabis, that's when I would go with more CBD. Because if they're not [consumed] to that intoxicated feel, then it could really turn them off to the medicinal – getting the medicinal benefits”
	Combined therapy with THC and CBD	“I have known cancer patients who followed this regime of using very high‐dose THC and CBD products for two months straight. You start with a very tiny bit of cannabis, a very light dose, and you move up to a very high dose. And it seems to really help people with ‐ just holistically. So somehow ‐ I don't know what the science is yet or what it's going to prove, but I think these super high doses of THC when combined with traditional treatments like chemo and radiation, I think that there's some special sauce there that makes both the traditional treatments more ‐ I don't know ‐ the body doesn't suffer as much, and it helps with tumor shrinkage also”
		“We have [Rick Simpson Oil] where it's a THC‐CBD. We have some that are one‐to‐ones. We have some that are two‐to‐ones… So, it all depends. I like to find out, from my customers, if they want to have that intoxicated feel, or if they don't want to have the intoxicated feel… The THC and that CBD combo provides the entourage effect, which is – provides a lot of medicinal benefits to help with easing their pain”
Hurdles in serving clients with cancer	Identifying clients with cancer at the dispensary counter	“I deal with patients on a normal basis, unless I look at their chart, I don't notice – I don't know any distinguishing factors of what their condition is. Unless I click on their chart and look at that, it's not like there's anything that specifically labels them as a cancer patient”
	Advising those who are THC‐naïve	“Rick Simpson, he has a whole regimen plan on how to take it, how much to take it. And I was able to dissect it based off of his plan and then some of the companies that make [Rick Simpson Oil] in town based off of their notes and their plan, and I kind of put both plans together. And it made a little scenario for somebody that's never [consumed] cannabis to [consume] it responsibly so they don't feel uncomfortable. You have to build it into your system until you're eventually doing one gram a day”
	Financial toxicity	“[This cancer patient] said that she was getting a great deal of relief even on the very small amount that she was able to [consume] because she didn't have a lot of money to spend. So we tried to make it as manageable as possible”
	Lack of standardized guidelines	“If we have a customer that comes in and asks about cancer, do you know how to handle that and assist them?… There's none of that… We had to figure that out on our own”
		“But I would say more so in when you have your onboarding with the company and you get that packet of information, I would say it goes through more of different cannabinoids and different terpenes and what each one is good for. But there isn't – there's not a page that says cancer, recommend X, Y and Z strain”

Interviews commenced with dispensary personnel describing indications for clients medicating with cannabis. Almost a third of participants described cancer as a very common reason (e.g., “top condition”, “half of… patients are cancer patients”). Interviewees reported that individuals with cancer turn to cannabis for four overarching reasons: (1) physical symptoms, (2) mental health symptoms, (3) disease modifying therapy, and (4) general wellbeing (e.g., “holistic,” “neuroprotectant potential,” “quality of life”). The cancer‐related physical symptoms referenced included poor appetite, nausea, vomiting, and pain (“neuropathic”). Mental health symptoms comprised anxiety (e.g., anticipatory, health, and existential), depression, and impaired sleep. As an example of such themes, one participant described:

For cancer patients, of course, appetite is a big one. Anxiety… anticipatory anxiety and nausea… neuropathy… sleep… We do have people who… think that it's a cancer cure.

A prominent theme was of cannabis's efficacy for cancer‐related symptoms and side‐effects. By contrast, there was little consensus among participants regarding the antineoplastic properties of cannabinoids. Of those who commented, most believed cannabis to be anecdotally efficacious, while some were steadfast believers in cannabis as cancer‐directed therapy. A few who considered cannabis to be anecdotally antineoplastic did not believe it the purview of dispensary staff to recommend cannabis for this indication. One stood in opposition to medicating with cannabis for cancer‐directed therapy due to the botanical's risk profile and worry that adults with cancer might forgo standard antineoplastic therapies to pursue cannabinoid‐based ones. A couple noted cannabis to cause fewer adverse effects than prescription opioid medications, chiefly because cannabis was viewed to be less obtunding.

Dispensary personnel described a client‐centered, symptom‐based approach to counseling adults with cancer. They denied the existence of a one‐size‐fits‐all oncologic cocktail. Interviewees referenced trial‐and‐error with regard to product selection and emphasized the necessity of understanding not just cancer symptom and treatment side‐effect severity, but which symptoms mattered most to a particular client. For instance, one dispensary employee commented:

It challenges you to come up with something that's unique and tailored to that individual and their needs versus just saying, ‘Oh, you have cancer. Okay, well, automatically, you must need this, this, and that because this is probably your symptoms.’ Even though they do have those as symptoms but they're not a big concern for that patient.

Interviewees cited edibles (e.g., cannabis‐infused baked goods and candies), sublingual tinctures, and high‐potency cannabinoid pastes (colloquially referred to as “Rick Simpson Oil” which is for the most part orally consumed) as both commonly suggested to and preferred by people facing cancer. For instance:

I think edibles are really great, and so are these really high‐potency pastes, for lack of a better word ‐ the THC and CBD pastes.

Of note, inhaled or sublingual tinctures were viewed as preferable when rapid relief was sought:

The quickest way of pain relief, the quickest onset of effect is going to be through inhalation.

However, an emergent theme was of individuals with cancer preferring to avoid inhalation. One participant articulated a multimodal approach:

A couple of puffs of your vape pen and then in about a half hour after… eat an edible. Okay? And the edible is going to kick in after… as your vape oil cartridge or your concentrate is going to start wearing off. So what you're doing is you're extending the relief; you're layering one on top of the other.

There was little consensus around which active ingredients to favor for individuals with cancer. A handful of participants recommended high potency delta‐9‐tetrahydrocannabinol (THC)‐predominant products; a few, high cannabidiol (CBD) ones; and a couple a progression from CBD‐predominant products to high THC‐predominant ones in THC‐naïve consumers. In general, it seemed that CBD was favored for those naïve to cannabinoids (to avoid THC‐related somnolence and paranoia), and high THC, for analgesia. For instance:

People that were already using cannabis or accepting of cannabis, then they want to have more intoxicated feeling. But for the ones that are going through treatment, don't know what else to do, they don't have any other options, their doctor said that they should try cannabis, that's when I would go with more CBD. Because if they're not used to that intoxicated feel, then it could really turn them off to getting the medicinal benefits.

A lot of [individuals with cancer] take high THC because… it tends to help with the pain.

Several challenges inherent in serving an oncologic population emerged in the interviews. First, dispensary personnel sometimes found it difficult to identify clients at the dispensary counter as having cancer:

Not all of our patients are upfront and disclose all of their ailments… I'm sure I've helped a lot of cancer patients but they just don't necessarily say it.

Second, this population may be THC‐naïve so there is often the necessity to start low with THC and go slow:

You start with a very tiny bit of cannabis, a very light dose, and you move up to a very high dose.

Third, this population may be financially burdened, limiting dosing power:

It's very heartbreaking when… a cancer patient that comes in and they're so weak and fragile and they need it, but they're not working like they used to have the money to buy enough of it. So you kinda have to… come up with some kind of plan for them to be able to get it and let them know the directions and how to take it and what to take so they won't spend as much money.

Fourth, unreliable product availability sometimes limits standardized dosing:

With the pandemic… we haven't been getting as much product as we used to. And for example, with the [Rick Simpson Oil]. I have one lady that I talk to five days a week that purchases the max amount of [Rick Simpson Oil] daily because she's going through cancer treatment and she's found that it's helped her so much. And we actually didn't have any for almost three/four weeks.

Fifth, standardized oncologic cannabis guidelines are absent at many levels of the system: in the oncology clinic, during dispensary onboarding procedures, and in client‐facing materials. For instance, participants reported:

The biggest thing that's lacking in the industry is evidence for me to say… you have cancer, this is what you need to use.

[There is a] need for clinical guidelines in oncology.

## 4 DISCUSSION

This paper represents one of the scientific community's first glimpses into dispensary staff views and advising practices around oncologic medical cannabis. Among participants, consensus seemed to have been reached about the botanical's efficacy for cancer‐related symptoms, less so regarding its antineoplastic potential. General principles for serving those with cancer included provision of client‐centered and symptom‐based care, for instance by tailoring a regimen according to a client's personal priorities and evolving that regimen based on feedback over time. This finding is of significance since on‐line recommendations for high concentration cannabinoid pastes often target a fixed high daily cannabinoid dose in a one‐size‐fits‐all fashion. Akin to traditional beliefs in medicine, non‐inhalation modes of administration were generally recommended to and preferred by individuals with cancer.^11^ An exception occurred when rapid relief from symptoms was desired. There was little consensus as to ratios of active ingredients optimal for adults with cancer. Some suggested that THC was not only more effective than CBD for symptoms such as pain, but also potentially more likely to cause euphoria and somnolence, and therefore to be consumed with caution in the cannabis‐naïve individual. In these instances, CBD‐predominant products would be preferable. Challenges in advising adults with cancer included being able to accurately identify them as such at the dispensary counter; arriving at a dosing regimen that was not cost‐prohibitive; and operating in the absence of clear oncologic guidelines.

While the informed assertions to come out of this study should be tested in a larger population‐based survey, if replicated, they would suggest that the oncologic community must grapple with the extent to which its members feel comfortable with both the nature and degree of counsel adults with cancer receive at the dispensary counter. The impressions and recommendations of dispensary personnel in this study seemed to mirror those of United States oncologists. In population‐based samples, oncologists largely agreed on the symptom management but not the antineoplastic potential of cannabis.^5^, ^12^, ^13^ Like dispensary personnel, they favored oral administration on net,^14^ but seemed not to have reached consensus on optimal ratios of cannabinoids.^15^ Given this philosophical alignment, clinicians who approve of the general direction of interactions at dispensaries may feel reassured. At the same time, a powerful theme through the transcripts was the degree to which dispensary staff, who are not uniformly trained, function in an almost clinical fashion: gathering history; recommending product formulations, ratios of active ingredients, route(s) of administration, and dosing schedules; and evolving those plans longitudinally based on client feedback.^7^, ^8^, ^9^ In some ways, the role of dispensary staff seemed to exceed that of a traditional pharmacist. For clinicians for whom this is a consideration, there may be impetus to increase involvement in guiding clinical conversations around oncologic cannabis. As two examples for possible concern: nearly absent from the transcripts was mention of drug–botanical interactions, and dispensary personnel in this study acknowledged that they are unlikely to have complete knowledge of diagnoses and concomitant medications given how the oncologic cannabis system is set up.

Data from this study also begin to suggest manners in which the oncologic cannabis system could be strengthened. First, insurance coverage for cannabis therapy would facilitate dosing consistency which may currently be cost prohibitive for some individuals with cancer. Second, formal dispensary guidelines for identifying and serving adults with cancer could serve to standardize an oncologic approach. As corollary, expanded on‐the‐job oncologic cannabis therapeutics training may be in order. Third, increased information transfer between medical facilities, which possess knowledge of diagnoses and concomitant medications, and dispensary storefronts, with grasp of the cannabis formulation and advice given, may improve the feedback loop for oncologic cannabis.^6^, ^16^

Our study has limitations. Its small convenience sample was vulnerable to selection bias. Potential bias could also have been introduced through use of multiple interviewers. Even in the face of these limitation, we believe that our diverse geographic, political, and regulatory sampling strengthened our informed assertions. Since socio‐behavioral research has demonstrated that oncology teams lean heavily on the brain trust of dispensary personnel to advise their patients. Understanding the advice given at the dispensary counter seems quite germane and stands to influence clinical care in this domain.^4^ Our exploratory study should be followed by a larger, in‐depth, oncological‐focused population‐based dispensary personnel survey, one that could serve as a valuable springboard to hypothesis generation in social psychology and clinical research pertaining to this important topic.

## AUTHOR CONTRIBUTIONS

**Manan M. Nayak:** Conceptualization (equal); data curation (lead); formal analysis (equal); investigation (equal); methodology (equal); project administration (equal); software (equal); supervision (equal); writing – original draft (equal); writing – review and editing (equal). **Peter R. Chai:** Data curation (equal); investigation (equal); methodology (equal); writing – original draft (supporting); writing – review and editing (supporting). **Stephanie Tung:** Writing – original draft (supporting); writing – review and editing (supporting). **James A. Tulsky:** Writing – original draft (supporting); writing – review and editing (supporting). **Marilyn J. Hammer:** Writing – original draft (supporting); writing – review and editing (supporting). **Nicole Andrade:** Writing – review and editing (supporting). **Ilana Braun:** Conceptualization (lead); data curation (equal); formal analysis (equal); funding acquisition (lead); investigation (equal); methodology (equal); project administration (equal); resources (equal); software (equal); supervision (equal); writing – original draft (equal); writing – review and editing (equal).

## FUNDING INFORMATION

Manan M Nayak, Peter R Chai, and Ilana M. Braun were funded by the Hans and Mavis Lopater Foundation; Peter R Chai was also funded by K23DA044847.

## CONFLICT OF INTEREST STATEMENT

Ilana Braun participated in a bench research project funded through a structured research agreement between Cannex Scientific and the Brigham and Women's Hospital. For all other authors there are no other conflict of interest.

## Data Availability

The data that support the findings of this study are available from the corresponding author upon reasonable request
